# BARRIERS AND FACILITATING FACTORS TO HEALTH MANAGEMENT TECH ADOPTION IN A RURAL MEALS ON WHEELS SAMPLE

**DOI:** 10.1093/geroni/igac059.3139

**Published:** 2022-12-20

**Authors:** David Wihry, Jennifer Jain, Jennifer Crittenden, Lenard Kaye, Tabatha Caso

**Affiliations:** University of Maine, Bangor, Maine, United States; University of Maine, Bangor, Maine, United States; University of Maine, Bangor, Maine, United States; University of Maine, Bangor, Maine, United States; Eastern Area Agency on Aging, Brewer, Maine, United States

## Abstract

Meals on Wheels clients served through the aging network represent a significant population of high need older adults who could benefit from health management technology. This poster presents the results of a study targeting Meals on Wheels clients in four rural U.S. counties on opportunities and barriers to adoption of technology-based health management interventions. Paper surveys were provided to 690 Meals on Wheels clients and 154 were returned (22.3% response rate). The mean age of respondents was 71.7 years and the sample was composed of 39% men and 61% women. When asked to select the most valuable features of home health management technology, 42% indicated vital signs monitoring, followed by remote access to classes and events at Area Agencies on Aging (21%), and video visits with medical providers (20%). When asked about current technology usage, 48% indicated never having engaged in a telehealth visit over a computer, smartphone or tablet. Top ranking barriers to health management technology utilization were affordability and privacy concerns. A majority (61%) of the sample disagreed or strongly disagreed that they have the money to afford a computer, smart phone, or tablet and 69% agreed or strongly agreed that they are concerned about privacy or scams when using the internet. Approximately four in ten (38%) are interested in using technology to manage their health. Additionally, 45% reported using a smartphone. The poster will also explore demographic differences in older consumer perceived barriers and opportunities and programmatic implications for expanding health management technology adoption.

